# Ethnic discrimination prevalence and associations with health outcomes: data from a nationally representative cross-sectional survey of secondary school students in New Zealand

**DOI:** 10.1186/1471-2458-12-45

**Published:** 2012-01-18

**Authors:** Sue Crengle, Elizabeth Robinson, Shanthi Ameratunga, Terryann Clark, Deborah Raphael

**Affiliations:** 1 Te Kupenga Hauora Māori, School of Population Health, Faculty of Medical and Health Sciences, University of Auckland, Private Bag 92019, Wellesley St, Auckland, New Zealand; 2 Epidemiology and Biostatistics, School of Population Health, Faculty of Medical and Health Sciences, University of Auckland, Private Bag 92019, Wellesley St, Auckland, New Zealand; 3 School of Nursing, Faculty of Medical and Health Sciences, University of Auckland, Private Bag 92019, Wellesley St, Auckland, New Zealand

## Abstract

**Background:**

Reported ethnic discrimination is higher among indigenous and minority adult populations. There is a paucity of nationally representative prevalence studies of ethnic discrimination among adolescents. Experiencing ethnic discrimination has been associated with a range of adverse health outcomes. NZ has a diverse ethnic population. There are health inequalities among young people from Māori and Pacific ethnic groups.

**Methods:**

9107 randomly selected secondary school students participated in a nationally representative cross-sectional health and wellbeing survey conducted in 2007. The prevalence of ethnic discrimination by health professionals, by police, and ethnicity-related bullying were analysed. Logistic regression was used to examine the associations between ethnic discrimination and six health/wellbeing outcomes: self-rated health status, depressive symptoms in the last 12 months, cigarette smoking, binge alcohol use, feeling safe in ones neighbourhood, and self-rated school achievement.

**Results:**

There were significant ethnic differences in the prevalences of ethnic discrimination. Students who experienced ethnic discrimination were less likely to report excellent/very good/good self-rated general health (OR 0.51; 95% CI 0.39, 0.65), feel safe in their neighbourhood (OR 0.48; 95% CI 0.40, 0.58), and more likely to report an episode of binge drinking in the previous 4 weeks (OR 1.77; 95% CI 1.45, 2.17). For all these outcomes the odds ratios for the group who were 'unsure' if they had experienced ethnic discrimination were similar to those of the 'yes' group.

Ethnicity stratified associations between ethnic discrimination and the depression, cigarette smoking, and self-rated school achievement are reported. Within each ethnic group participants reporting ethnic discrimination were more likely to have adverse outcomes for these three variables. For all three outcomes the direction and size of the association between experience of ethnic discrimination and the outcome were similar across all ethnic groups.

**Conclusions:**

Ethnic discrimination is more commonly reported by Indigenous and minority group students. Both experiencing and being 'unsure' about experiencing ethnic discrimination are associated with a range of adverse health/wellbeing outcomes. Our findings highlight the progress yet to be made to ensure that rights to be free from ethnic discrimination are met for young people living in New Zealand.

## Background

Racism, the belief that some population groups ('races' or ethnic groups) are superior to others, ensures that power and privilege within society is conferred on the group(s) considered superior [1,2]. Ethnic discrimination, the behavioural manifestation of racism, is defined as "unfair, differential treatment on the basis of race or ethnicity" [3]. Discriminatory acts between individuals constitute interpersonal discrimination while discriminatory policies or practices embedded in organisational structures constitute institutional discrimination [4-6].

The majority of ethnic discrimination research has been undertaken in adult populations. Among adult populations in the US, UK, Australia and New Zealand, reported ethnic discrimination is higher among indigenous and minority populations [4,7-10]. There is a paucity of nationally representative prevalence studies of ethnic discrimination among adolescents. Experiencing ethnic discrimination has been associated with a range of adverse physiological, physical, psychological, and behavioural outcomes among adult and adolescent populations including: poor self-reported overall health status [4,11-14]; depression, somatic complaints, anxiety and psychosis [3,7,12-21]; and risk-taking behaviours such as smoking, increased alcohol consumption, and use of other psychoactive substances [14,20-23]. Experiencing discrimination has also been shown to be associated with ethnic disparities in access to, and quality of care [14]. In NZ, self-reported discrimination has been found to account for some of the observed health inequalities between Māori and NZ European adults [24].

New Zealand has a diverse population. The 2006 population census enumerated 606207 people aged 10-19 years. Māori (the indigenous people of New Zealand) accounted for 20.7% of this age group, with 9.8% identifying Pacific ethnic groups, 9.9% Asian ethnic groups, 62.4% European and 9.0% as other ethnic group. Note that percentages add up to more than 100 as people who identified with more than one ethnic group have been counted in all ethnic groups they identified.

New Zealand youth health statistics leave much to be desired with rates of suicide, death from motor vehicle crashes, and alcohol and other substance use among some of the highest in the Western world [25]. Furthermore, there are significant ethnic differences in health and wellbeing outcomes. Compared to non-Māori, Māori youth aged 15-24 years have significantly higher mortality rates from accidents and suicide [26] and significantly higher rates of hospitalisation for injury/poisoning and mental/behavioural disorders [27]. Greater proportions of Pacific and Māori secondary school students reported smoking cigarettes at least weekly, using marijuana at least weekly, and having made a suicide attempt in the previous 12 months [28,29].

The purpose of this paper is to contribute to the literature about ethnic discrimination among adolescents by reporting the findings from a nationally representative survey of young people attending secondary school in New Zealand. We describe the self-reported prevalence of experience of ethnic discrimination and the associations between ethnic discrimination and health and wellbeing outcomes.

## Methods

*Youth'07 The Second National Health and Wellbeing Survey of New Zealand Secondary School Students *(Youth'07) is a nationally representative cross-sectional survey of the health and wellbeing of Year 9 to 13 secondary school students in New Zealand (NZ) conducted in 2007. The study design, described in detail elsewhere [30], is summarised here. A two-stage clustered sampling design was used. One hundred and fifteen randomly selected schools were invited to participate and, in each school, students were randomly selected and invited to participate. The response rate for schools was 84% and for students was 74%. In total, 9107 students completed the survey, accounting for 3.4% of the total number of students enrolled in secondary schools in 2007. All data were collected during school days, in designated rooms in each school using Multimedia Computer Assisted Self Interview (M-CASI). Each student received a login code to access the anonymous 622 item multimedia survey which was self-administered using hand-held internet tablets [31]. Written consent to participate was obtained from each participating school. Written information about the study was given to the selected students and their parents. On the day of the survey each student provided consent to participate. The University of Auckland Human Subjects Ethics Committee approved the study.

Self-reported ethnicity data was collected according to the Statistics New Zealand standard for the electronic collection of ethnicity data [32]. Students were able to choose multiple ethnic groups. Where this was the case ethnicity data were categorised into a single ethnic group using a hierarchical prioritisation method that prioritised ethnicity in the following order: Māori, Pacific, Asian, Other, or NZ European [33]. Māori are the indigenous people of NZ. Pacific ethnic groups include Samoan, Tongan, Niuean, Cook Island Māori and other Pacific Islands. The Other ethnic group compromises all ethnic groups that are not included in the Māori, Pacific, Asian and NZ European categories. The Asian ethnic group includes participants from Asia and the Indian sub-continent. The 'other' ethnic group is extremely diverse and includes participants from Middle East, Africa, South America, North America, and counties in Europe.

Three socio-economic variables were included in the analyses: the New Zealand Index of Deprivation 2006 (NZDep2006) decile [34], food security, and housing mobility. NZDep2006 deciles were categorised into three deprivation groups: low (Index deciles 1-3), moderate (4-7), and high deprivation (8-10). Food security was assessed by asking 'Do your parents, or the people who act as your parents, ever worry about not having enough money to buy food?' and responses were categorised into 'never', 'occasionally/sometimes', and 'often/all the time'. Housing mobility was assessed by asking 'In the past year how many times have you moved homes?' and responses were dichotomised into 'none or one time' and 'two or more times'. Age was categorised into five discrete levels: ≤ 13 years, 14, 15, 16 and ≥ 17 years.

Students' experiences of ethnic discrimination in three settings were examined: unfair treatment by the police because of ethnicity, unfair treatment by health professionals because of ethnicity, and bullying because of ethnicity. Response options for the unfair treatment variables were 'no', 'yes', and 'unsure' and the time period was 'ever' experienced these types of discrimination. Participants who reported they had been bullied were asked to indicate the reason(s) for bullying from a drop-down list that included 'I was bullied because of my ethnic group or culture'. The timeframe for 'ethnicity-related bullying' is 'in the current school year'. A summary variable, 'any ethnic discrimination', was developed to assess the overall experience of ethnic discrimination.

Six measures were used to assess the impact of self-reported discrimination on health and wellbeing: self-rated health status, depressive symptoms in the last 12 months, cigarette smoking at least weekly, binge alcohol use in the previous 4 weeks, feeling safe in ones neighbourhood, and self-rated achievement at school. Self-rated health was dichotomised into 'excellent/very good/good' and 'fair/poor'. The Reynolds Adolescent Depression Scale (RADS) which measures depressive symptomatology in young people aged 12 to 18 years [35], has been validated for use in the NZ population both in its original [36] and in the short form [37]. Assessment of the short form version (RADS-SF) found that a cut off of 28 for depressive symptoms gave best agreement with the prevalence of depressive symptoms determined by the long form of the RADS. Accordingly we have used a RADS-SF score of ≥ 28 to identify students with significant depressive symptoms [37]. Cigarette smoking was categorised into students who smoked at least weekly and those who had never smoked or smoked less than once per week. Binge drinking was defined as having five or more alcoholic drinks in one session within a 4 hour period. The binge drinking variable was categorised as students who did not drink binge drink in the previous 4 weeks and those who had at least one episode of binge drinking in the previous 4 weeks. Students were asked if they felt safe in their neighbourhood and data were dichotomised into 'feeling safe all/most of the time' and 'sometimes/mostly not/not feeling safe'. Self-rated achievement at school was assessed by asking students how well they did at school with dichotomisation of responses into 'near the top/above the middle/about the middle' and 'below the middle/near the bottom'.

Only participants with data on discrimination variables have been included in the analyses. No imputation has been done for missing variables. Data was analysed using Statistical Analysis Software (Version 9.2; SAS Institute, North Carolina, USA). All analyses were adjusted for design effects (clustering and unequal probability of selection). Descriptive data analyses (frequency distributions, 95% confidence intervals, Chi-square analyses) were stratified by ethnicity and by discrimination status. Logistic regression modelling was employed to analyse the associations between ethnicity and ethnic discrimination variables and the associations between ethnic discrimination and health/wellbeing outcome variables while adjusting for confounding variables. New Zealand European students were used as the reference group in all logistic regression analyses.

## Results

Ethnicity data were available for 9080 of the 9107 participating students. NZ European was the most frequently reported ethnic group (53%) followed by Māori (19%), Asian (13%), Pacific (10%) and other ethnic groups (6%). The median age was 14 years with approximately 99% of students aged between 13 and 18 years and about 1% aged less than 13 years or over 18 years of age (data not shown). There were significant ethnic differences in age distribution with lower proportions of Māori, Pacific and Other ethnic groups in the older ages. Significant ethnic differences in socioeconomic variables were observed with higher proportions of Pacific and Māori students living in high deprivation areas, reporting food insecurity, and reporting housing mobility. (Table 1)

**Table 1:** Sociodemographic data by ethnicity from Youth'07-national survey of secondary school student's health and wellbeing, New Zealand 2007

	Māori		Pacific		Asian		Other		NZ European		Total	
	n	%	n	%	n	%	n	%	n	%	n	%
		95% CI		95% CI		95% CI		95% CI		95% CI		95% CI
Total	1702		924		1126		531		479		9080	
		18.7		10.2		12.5		5.9	7	52.6		
		15.6-21.9		6.4 - 14.1		7.6 - 17.4		5.0 - 6.8		47.6 - 57.7		

Sex												
Male	882	52.0	497	53.8	619	55.1	273	51.3	263	55.0	4902	54.1
		45.6 - 58.4		43.7 - 63.9		42.8 - 67.4		43.7 - 58.9	1	47.4 - 62.5		47.5 - 60.7
Female	820	48.0	427	46.2	507	44.9	258	48.7	216	45.0	4178	45.9
		41.6 - 54.4		36.1 - 56.3		32.6 - 57.2		41.1 - 56.3	6	37.5 - 52.6		39.3 - 52.5

Age (years)												
≤ 13	414	24.1	213	23.0	163	14.5	107	20.1	959	19.8	1856	20.3
		21.3 - 26.8		20.6 - 25.5		10.5 - 18.4		16.6 - 23.5		17.8 - 21.9		18.5 - 22.1
14	434	25.6	201	21.6	211	18.7	109	20.3	113	23.7	2094	23.0
		23.2 - 27.9		19.1 - 24.2		16.8 - 20.7		16.0 - 24.6	9	22.2 - 25.2		21.7 - 24.3
15	387	22.8	196	21.3	221	19.5	133	25.2	103	21.6	1968	21.7
		20.9 - 24.7		18.5 - 24.1		17.8 - 21.2		21.1 - 29.3	1	20.4 - 22.8		20.9 - 22.6
16	268	15.8	182	19.7	252	22.4	107	20.2	932	19.5	1741	19.2
		14.2 - 17.5		17.1 - 22.4		19.6 - 25.3		17.2 - 23.2		18.1 - 20.8		18.1 - 20.3
≥ 17	199	11.7	132	14.3	279	24.9	75	14.2	736	15.4	1421	15.7
		10.1 - 13.3		11.9 - 16.7		21.8 - 27.9		10.3 - 18.1		13.9 - 16.9		14.3 - 17.1

NZDep2006												
Low deprivation	334	20.3	70	7.8	424	38.6	219	42.4	216	46.4	3215	36.4
		16.3 - 24.2		4.6 - 11.0		29.6 - 47.5		33.3 - 51.5	8	41.6 - 51.3		31.1 - 41.7
Medium deprivation	577	34.9	221	24.8	481	43.8	190	36.9	192	41.0	3395	38.3
		30.9 - 38.9		18.2 - 31.4		39.8 - 47.8		31.6 - 42.2	6	37.7 - 44.4		35.5 - 41.2
High deprivation	743	44.9	601	67.4	194	17.6	107	20.7	594	12.5	2239	25.3
		38.3 - 51.4		58.4 - 76.4		9.4 - 25.9		13.9 - 27.5		10.2 - 14.9		19.8 - 30.7

Parents worry about having enough money to buy food												
Never	869	54.8	362	43.5	740	71.2	362	43.5	311	70.1	5415	64.4
		52.3 - 57.4		38.6 - 48.4		66.6 - 75.7		38.6 - 48.4	8	67.7 - 72.6		61.5 - 67.4
Occasionally/sometimes	553	34.7	336	40.4	233	22.2	336	40.4	109	24.3	2352	27.8
		32.2 - 37.2		36.0 - 44.9		19.0 - 25.4		36.0 - 44.9	4	22.4 - 26.3		25.6 - 29.9
Often/all the time	165	10.5	134	16.1	70	6.6	134	16.1	251	5.6	659	7.8
		8.6 - 12.4		13.8 - 18.4		4.6 - 8.6		13.8 - 18.4		4.8 - 6.4		6.7 - 8.8

Moved home 2 or more times in last 12 months												
Yes	311	18.7	187	20.7	177	16.0	70	13.5	393	8.3	1138	12.8
		16.9 - 20.4		16.2 - 25.2		13.5 - 18.4		10.7 - 16.4		7.4 - 9.2		11.7 - 13.9
No	1362	81.3	718	79.3	933	84.0	451	86.5	433	91.7	7794	87.2
		79.6 - 83.1		74.8 - 83.8		81.6 - 86.5		83.6 - 89.3	0	90.8 - 92.6		86.1 - 88.3

Ethnicity-related bullying was most commonly reported by Asian participants. Māori, Pacific and Other ethnic groups were also significantly more likely to report this experience than the NZ European ethnic group. Pacific, Māori, Asian and Other ethnic groups were significantly more likely to report experiencing ethnic discrimination by the police than NZ European participants. Māori, Pacific and Asian participants were significantly more likely to report being unsure if they had experienced ethnic discrimination by the police than NZ European participants. Pacific, Asian, Māori and Other ethnic groups were significantly more likely to report ethnic discrimination and being unsure about experiencing ethnic discrimination by health professionals than NZ European participants. Experiencing and being unsure about experiencing discrimination in one or more of the three dimensions ('any ethnic discrimination') were significantly more common among Asian, Pacific, Māori and Other ethnic groups than NZ European. (Table 2)

**Table 2:** Ethnic discrimination, crude prevalences by ethnicity and adjusted comparisons between ethnic groups, Youth'07

			Maori	Pacific	Asian	Other	NZ European	Total
		n	52	31	95	19	83	280
Had experienced ethnicity-related bullying N = 8821		%	3.1	3.5	8.5	3.7	1.8	3.2
	Yes	95% CI	2.3 - 4.0	2.2 - 4.8	6.8 - 10.2	1.7 - 5.8	1.3 - 2.3	2.7 - 3.7
		OR	1.61	1.61	5.36	2.28		
		95% CI	1.01 - 2.56	0.99 - 2.62	3.75 - 7.67	1.27 - 4.12	1	

		n	96	58	57	20	99	330
		%	6.5	7.5	5.4	4.2	2.2	4.0
	Yes	95% CI	5.1 - 7.8	5.0 - 10.1	3.6 - 7.2	2.5 - 5.9	1.7 - 2.7	3.4 - 4.6
Treated unfairly by the police because of ethnicity N = 8284		OR	2.78	3.10	2.70	2.03		
		95% CI	2.03 - 3.80	2.02 - 4.73	1.75 - 4.16	1.25 - 3.32	1	
								
		n	128	69	63	16	128	404
		%	8.6	9.0	6.0	3.2	2.8	4.9
	Unsure	95% CI	7.1 - 10.1	7.1 - 10.9	4.6 - 7.4	2.0 - 4.5	2.3 - 3.4	4.2 - 5.5
		OR	3.10	2.77	2.27	1.22		
		95% CI	2.30 - 4.17	1.86 - 4.14	1.63 - 3.17	0.78 - 1.91	1	

		n	84	74	58	19	95	330
		%	5.0	8.6	5.3	3.7	2.0	3.8
	Yes	95% CI	4.1 - 6.0	6.7 - 10.4	4.0 - 6.5	1.9 - 5.6	1.5 - 2.6	3.2 - 4.3
Treated unfairly by health professional because of your ethnicity N = 8785		OR	2.41	3.82	3.17	2.03		
		95% CI	1.76 - 3.31	2.63 - 5.57	2.03 - 4.95	1.09 - 3.76	1	
								
		n	248	117	230	73	386	1054
		%	15.0	13.5	20.9	14.4	8.3	12.0
	Unsure	95% CI	13.4 - 16.6	10.9 - 16.1	17.9 - 23.8	10.9 - 17.9	7.3 - 9.2	11.0 - 13.0
		OR	1.57	1.38	3.27	1.79		
		95% CI	1.32 - 1.86	1.07 - 1.78	2.76 - 3.89	1.30 - 2.45	1	

		n	197	139	180	52	238	806
		%	12.9	17.4	16.7	10.8	5.4	9.7
	Yes	95% CI	11.0 - 14.8	14.2 - 20.7	14.0 - 19.4	7.8 - 13.7	4.5 - 6.2	8.6 - 10.7
		OR	2.49	3.18	4.54	2.48		
Any ethnic discrimination N = 8345		95% CI	1.96 - 3.16	2.34 - 4.32	3.49 - 5.91	1.82 - 3.38	1	
								
		n	290	136	220	79	431	1156
		%	19.1	17.0	20.6	16.3	9.6	13.9
	Unsure	95% CI	16.7 - 21.5	14.2 - 19.8	17.5 - 23.6	12.5 - 20.0	8.6 - 10.7	12.7 - 15.1
		OR	1.92	1.61	3.09	1.88		
		95% CI	1.58 - 2.33	1.24 - 2.07	2.60 - 3.69	1.40 - 2.51	1	

Odds ratios compare the odds of reporting ethnic discrimination for each ethnic group using NZ European as the reference group and are adjusted for age, sex, NZDep2006, food security, housing mobility

In unadjusted bivariate analyses, self-reported experience of ethnic discrimination in each of the three dimensions and 'any ethnic discrimination' were related to adverse outcomes for all of the health and well-being variables under investigation. (Table 3).

**Table 3:** Health and wellbeing outcomes by ethnic discrimination, Youth'07

			General excellent/very good/good	RADS significant depression symptoms	Smokes cigarettes at least weekly	Binge drinking in last 4 weeks	Feels safe in neighbourhood all/most of time	Self-rated achievement in school near top/above or about the middle
	
Had experienced ethnicity-related bullying	Yes	n	241	64	26	78	168	244
		%	86.5	23.6	9.7	30.0	65.3	87.1
		95% CI	82.3 - 90.7	17.7 - 29.5	5.8 - 13.5	23.8 - 36.3	58.7 - 71.9	83.4 - 90.8
	
	No	n	7832	881	612	2738	6511	7807
		%	92.1	10.5	7.7	34.7	81.7	91.5
		95% CI	91.3 - 92.9	9.6 - 11.4	6.8 - 8.5	32.1 - 37.3	80.0 - 83.3	90.7 - 92.3
Treated unfairly by the police because of ethnicity	Unsure	n	345	97	64	168	255	343
		%	85.2	23.9	17.5	47.2	64.9	84.8
		95% CI	81.7 - 88.8	19.8 - 28.1	14.0 - 21.0	40.9 - 53.6	60.3 - 69.4	81.5 - 88.0
	
	Yes	n	278	67	72	178	255	271
		%	84.4	20.7	23.7	56.8	64.9	82.3
		95% CI	80.3 - 88.6	16.6 - 24.9	18.7 - 28.6	50.0 - 63.5	60.3 - 69.4	78.5 - 86.1
	
	No	n	7006	732	468	2397	6191	6986
		%	92.8	9.7	6.3	32.5	82.5	92.6
		95% CI	92.1 - 93.5	8.8 - 10.6	5.5 - 7.1	29.8 - 35.2	80.9 - 84.1	91.8 - 93.4

Treated unfairly by a health professional because of ethnicity	Unsure	n	922	201	94	309	695	914
								
		%	87.6	19.3	9.6	32.8	72.2	86.7
		95% CI	85.5 - 89.7	16.7 - 21.9	7.8 - 11.5	29.4 - 36.2	68.9 - 75.5	84.3 - 89.1
	
	Yes	n	273	70	46	120	188	285
		%	83.1	22.2	15.9	41.3	66.4	86.9
		95% CI	78.3 - 87.9	17.7 - 26.7	11.7 - 20.1	34.7 - 48.0	61.1 - 71.8	82.9 - 90.9
	
	No	n	6876	684	502	2393	5828	6825
		
		%	92.9	9.3	7.1	34.4	83.0	92.4
		95% CI	92.1 - 93.7	8.4 - 10.3	6.3 - 8.0	31.7 - 37.2	81.4 - 84.6	91.6 - 93.2

Any ethnic discrimination	Unsure	n	1020	207	112	359	758	1000
		%	88.2	18.1	10.7	35.0	71.1	86.4
		95% CI	86.2 - 90.3	15.8 - 20.5	8.9 - 12.4	31.2 - 38.8	68.0 - 74.2	84.1 - 88.7
	
	Yes	n	689	164	111	304	505	696
		%	85.8	20.9	15.0	41.3	69.2	86.5
		95% CI	83.1 - 88.4	17.9 - 23.9	12.0 - 17.9	36.6 - 46.0	66.1 - 72.4	84.2 - 88.8
	
	No	n	5958	542	384	2094	5348	5946
		%	93.3	8.5	6.1	33.5	84.2	93.2
		95% CI	92.6 - 94.1	7.5 - 9.4	5.3 - 6.9	30.7 - 36.3	82.6 - 85.8	92.5 - 94.0

Logistic regression analysis was used to explore the relationship between experiencing 'any ethnic discrimination' and the health and wellbeing outcomes after adjusting for age, sex, ethnicity and the three socioeconomic variables. Testing for interaction between ethnicity and 'any ethnic discrimination' was undertaken for all outcome variables. As there was no significant interaction for the general health, binge drinking and feeling safe in the neighbourhood variables the results of logistic regressions are reported for the total sample. Compared with participants who did not experience ethnic discrimination, those who reported 'yes' or 'unsure' were significantly less likely to report excellent/very good/good self-rated general health, less likely to feel safe in their neighbourhood, and more likely to have had an episode of binge drinking in the previous four weeks. (Table 4).

**Table 4:** Logistic regressions for associations between experiencing ethnic discrimination and general health, binge drinking and feeling safe in the neighbourhood outcomes and, Youth'07

	Experienced any form of discrimination		
	Unsure OR 95% CI	Yes OR 95% CI	No OR 95% CI
General health excellent/very good/good	0.61 0.47 - 0.78	0.51 0.39 - 0.65	1.0

Binge drinking in last 4 weeks	1.42 1.20 - 1.68	1.77 1.45 - 2.17	1.0

Feels safe in neighbourhood all/most of the time	0.59 0.51 - 0.68	0.48 0.40 - 0.58	1.0

Adjusted for ethnicity, age, sex, NZDep2006, food security, housing mobility

Significant interactions between ethnicity and 'any ethnic discrimination' were observed for the depression (*p *= 0.009), smoking cigarettes (*p *= 0.04), and school achievement (*p *= 0.016) variables. Stratification resulted in loss of precision around the odds ratio point estimates in all ethnic groups. Within each ethnic group participants who reported 'yes' or 'unsure' experience of ethnic discrimination were more likely to have experienced significant depressive symptoms and smoke cigarettes at least weekly and less likely to rate their school achievement 'near top/above or about the middle' than those who had not experienced ethnic discrimination. For all three outcomes the direction and size of the association between experience of ethnic discrimination and the outcome were similar across all ethnic groups. (Table 5)

**Table 5:** Ethnicity stratified logistic regressions for associations between experiencing ethnic discrimination and depression, smoking and school achievement outcomes, Youth'07

	Māori	Pacific	Asian	Other	NZ European
Significant depressive symptoms in past 12 months					
Yes					
OR	3.35	2.94	2.28	2.77	4.10
95% CI	1.98, 5.67	1.53, 5.64	1.55, 3.36	1.19, 6.45	2.90, 5.80
Unsure					
OR	1.94	1.93	2.53	1.08	2.32
95% CI	1.24, 3.04	1.07, 3.48	1.67, 3.83	0.59, 1.99	1.69, 3.18
No	1.0	1.0	1.0	1.0	1.0

Smokes cigarettes at least weekly					
Yes					
OR	3.62	1.29	1.50	3.37	4.00
95% CI	2.31, 5.67	0.62, 2.60	0.62, 3.64	0.93, 12.25	2.62, 6.10
Unsure					
OR	1.76	0.96	1.15	5.47	2.05
95% CI	1.16, 2,68	0.35, 2.60	0.48, 2.78	2.23, 13.43	1.42, 2.96
No	1.0	1.0	1.0	1.0	1.0

Achievement near top/above or about the middle					
Yes					
OR	0.44	0.77	0.93	0.37	0.52
95% CI	0.29, 0.67	0.37, 1.61	0.47, 1.85	0.14, 0.97	0.35, 0.77
Unsure					
OR	0.71	0.73	0.39	0.21	0.51
95% CI	0.49, 1.05	0.42, 1.26	0.20, 0.77	0.10, 0.43	0.35, 0.74
No	1.0	1.0	1.0	1.0	1.0

Adjusted for age, sex, NZDep2006, food security, housing mobility

## Discussion

This large, nationally representative study provides prevalence estimates of secondary school student's experience of ethnicity-related bullying at school and their experience of ethnic discrimination by health professionals and by the police. In doing so, it contributes to a limited literature about the effect of experiencing ethnic discrimination on the health and wellbeing of young people. It also contributes to a very limited evidence base regarding the effects of ethnic discrimination on the health of indigenous youth.

Māori, Pacific, Asian and Other ethnic group participants were significantly more likely to report experiencing ethnic discrimination than their NZ European peers. Furthermore, the reporting of being unsure about ethnic discrimination was significantly more likely among Māori, Pacific, Asian and Other ethnic groups than the NZ European ethnic group.

Participants who reported 'yes' or 'unsure' to the questions on ethnic discrimination were significantly less likely to report excellent/very good/good health, feel safe in their neighbourhood, and report their achievement at school as about the middle or higher. These participants were also significantly more likely to: have experienced depressive symptoms in the previous 12 months, smoke cigarettes at least weekly, and to have had an episode of binge drinking in the previous 4 weeks. The adverse associations between being 'unsure' about ethnic discrimination and health/wellbeing outcomes were remarkably similar to those who reported 'yes' to experiencing this ethnic discrimination. Our results suggest that for both 'yes' and 'unsure' groups ethnic discrimination may be an important determinant of health and wellbeing.

Youth/school student experience of ethnic discrimination by a health professional has not been, to our knowledge, reported in the literature. However, our findings are consistent with those of the 2002/2003 NZ health survey (participants ≥ 15 years of age) which found that Māori, Pacific, and Asian adults were significantly more likely to report unfair treatment by a health professional than NZ Europeans [4] and with the international literature that reports that adults from ethnic minorities are more likely to experience differential and unfair treatment by health professionals than dominant ethnic groups [38-41]. Our findings that Pacific, Māori, Asian and Other ethnic group's participants were significantly more likely to report ethnic discrimination by the police is consistent with international literature that shows youth from minority ethnic groups are most likely to report ethnic discrimination or harassment by the police [42-44].

Compared with NZ European students, all other ethnic groups in the current study were more likely to report being bullied at school because of their ethnicity/culture, with Asians having the highest self-report rates of all groups. The Youth2000 survey also found that Asian students were the ethnic group most likely to report ethnicity related bullying in schools, with Pacific then Maori students the next highest groups [45]. A NZ survey of international students (92% of whom were Asian and most were at secondary school), revealed that less than half believed that New Zealanders have positive attitudes towards international students and a third believed that ethnic discrimination was a common experience among international students [46]. The studies noted above were not designed to explore the reasons for these findings. However, a small pilot study of secondary school students in New Zealand suggests that inter-ethnic 'intimidatory practices' experienced by Asian ethnic minorities may reflect social distance (separation) between Asian and non-Asian ethnic groups and/or the Asian ethnic groups' perceptions about acceptance in the wider community [47]. These issues require further exploration alongside consideration of potential intersecting issues such as the experience of being (in some cases) newer migrants [48]. Literature from the US and the UK also document the prevalence and severity of ethnicity-related bullying among minority ethnic groups in comparison to the dominant ethnic group [49,50].

In NZ, Harris et al. [4] found that adults who reported experiencing ethnic discrimination were significantly more likely to report poor/fair self-rated health, lower physical functioning, poorer mental health, and current smoking. Our findings amongst secondary students were similar, with students who reported ethnic discrimination being more likely to report fair/poor self-rated health, have experienced significant depressive symptoms, and be cigarette smokers. Our findings are also consistent with international reports that, among adult and youth populations, ethnic discrimination is associated with negative self-reported health and poorer overall health status [9,11-14,51], depressive symptoms or depression [7,13,14,20,52-55], smoking [14,20-22,56-58], alcohol use [14,20-22], and use of other substances [14,20,21,23]. International literature has also reported that youth who experience ethnic discrimination are more likely to feel unsafe in their neighbourhood because of bullying that started in school and overflowed into the victims' neighbourhood [59] and that those who experience ethnicity-related bullying in schools are more likely to have lower levels of academic achievement [60,61]. While our study used 'any ethnic discrimination' rather than ethnicity-related bullying specifically, the international conclusions are consistent with our findings.

Due to the cross-sectional design of the study we are able to report associations but not attribute causality. Other literature discusses hypothesised mechanisms and pathways between experiencing ethnic discrimination and adverse health outcomes [14,55]. Williams and Mohammed [14] describe three potential pathways through which experiencing ethnic discrimination may adversely affect health. They argue that exposure to stress (ethnic discrimination) results in psychological distress that adversely affects health; that behavioural coping strategies to manage stress may include unhealthy behaviours such as smoking and alcohol misuse; and that psychological and behavioural responses to stressors can lead to structural and functional alterations in physiological systems [14]. Personal and social factors such as the strategies used to cope with stressors, social support, level of vigilance and anticipatory anxiety about ethnic discrimination, ethnic identity, and ethnic group identification may also moderate or mediate the effect of experiencing ethnic discrimination on health outcomes [14,55]. Bals, Turi, Skre and Kvernmo [62] found that 'enculturation factors' such as participation in cultural activities and Sami language competence were associated with decreased mental health problems among Indigenous Sami youth. The interactions between enculturation/cultural resilience, experiencing ethnic discrimination and health outcomes require further elucidation.

This study has a number of strengths: it is a large, nationally representative survey with good school and student response rates. The anonymous survey using M-CASI allowed investigation of sensitive issues that may be under-reported using other data collection methods such as face-to-face interviewing.

There are a number of limitations that should be considered. Firstly, the survey was conducted with young people attending secondary schools. The experiences and health outcomes among young people who are not attending school may systematically differ from those who are attending school [63]. In addition, ethnic discrimination has been described as a significant influence on early school leavers [64]. Māori and Pacific youth in NZ leave school early [65,66] suggesting that the findings in this study may underestimate the prevalence of ethnic discrimination among the 'total' (i.e. including those that are not at school) population of young people in this age group.

Secondly, participants were asked about their experience of ethnicity-related bullying and ethnic discrimination by health professionals and the police. There are a number of other domains where participants may have been exposed to ethnic discrimination, for example by staff at schools and in other social settings, which have not been included in the current study. Furthermore, institutional forms of discrimination are unlikely to have been captured by the measures used in the current study. As a result our findings may underestimate the overall experience of ethnic discrimination experienced by participants.

Thirdly, some data may be vulnerable to recall bias. As the accuracy of recall is unlikely to be influenced by the participant's ethnic group, or their experience of ethnic discrimination, it is unlikely that recall bias will substantially alter the effect estimates. Fourthly, the measurement of socio-economic position among adolescents poses challenges as they may be unable to provide accurate information about common measures of socio-economic position such as parental occupation, education level, or income [67,68]. This study used three alternative socio-economic variables; however, there may still be residual confounding by socio-economic position.

Finally, we are unable to ascertain whether students who responded they were 'unsure' were unsure whether they had experienced discrimination, or were unsure whether this was because of their ethnicity. 'Unsure' responses may reflect attributional ambiguity where the experience is ambiguous and difficult for recipients to label as discriminatory [14].

Future research could address ethnic discrimination in the school setting, non-bullying ethnic discrimination by peers, and the nature, frequency and intensity of discriminatory experiences. In addition, studies are needed to clarify the nature of 'unsure' responses and to examine the associations between ethnic discrimination and health/wellbeing outcomes with a view to understanding the pathways between experiencing ethnic discrimination and adverse health and wellbeing outcomes and potential interventions.

## Conclusions

Freedom from ethnic discrimination is embedded with the UN Declaration of Human Rights, the NZ Bill of Rights Act, and other international human rights instruments. In conjunction with the findings relating to the adult population by Harris et al., our findings that secondary school students are cognisant of ethnic discrimination speak to the progress yet to be made in wider society to ensure that these rights are met for all people living in Aotearoa New Zealand.

## Abbreviations

NZ: New Zealand; NZDep2006: New Zealand Index of Deprivation 2006; RADS: Reynolds Adolescent Depression Scale; RADS-SF: Reynolds Adolescent Depression Scale short form version; US: United States of America; UK: United Kingdom.

## Competing interests

The authors declare that they have no competing interests.

## Authors' contributions

SC was involved in the conception and design, acquisition of data, data analysis and interpretation, wrote this manuscript, and has given final approval of the version. ER was involved in the conception and design, acquisition of data, data analysis and interpretation, revised the manuscript critically for important intellectual content, and has given final approval of the version. SA was involved in the conception and design, acquisition of data, data analysis and interpretation, revised the manuscript critically for important intellectual content, and has given final approval of the version. TC was involved in the conception and design, acquisition of data, data analysis and interpretation, revised the manuscript critically for important intellectual content, and has given final approval of the version. DR was involved in data interpretation, drafting of the manuscript, revised the manuscript critically for important intellectual content, and has given final approval of the version.

## Authors' information

SC is a Senior Lecturer (Medical) and Director of Tōmaiora Māori Health Research Centre. ER is a Senior Research Fellow. SA is a Professor of Epidemiology. TC is a Senior Lecturer in the School of Nursing. DR is a Research Assistant in the School of Nursing.

## Pre-publication history

The pre-publication history for this paper can be accessed here:

http://www.biomedcentral.com/1471-2458/12/45/prepub

## References

[B1] Bhopal R Spectre of racism in health and health care: lessons from history and the United States Br Med J 1998 316 1970 1973 10.1136/bmj.316.7149.1970 PMC1113412 9641943

[B2] Williams DR Race and health: basic questions, emerging directions Ann Epidemiol 1997 7 322 333 10.1016/S1047-2797(97)00051-3 9250627

[B3] Greene ML Way N Pahl K Trajectories of perceived adult and peer discrimination among Black, Latino, and Asian American adolescents: patterns and psychological correlates Dev Psychol 2006 42 218 236 16569162 10.1037/0012-1649.42.2.218

[B4] Harris R Tobias M Jeffreys M Waldegrave K Karlsen S Nazroo J Racism and health: The relationship between experience of racial discrimination and health in New Zealand Soc Sci Med 2006 63 1428 1441 10.1016/j.socscimed.2006.04.009 16740349

[B5] Krieger N Embodying inequality: a review of concepts, measures, and methods for studying health consequences of discrimination Int J Health Serv 1999 29 295 352 10.2190/M11W-VWXE-KQM9-G97Q 10379455

[B6] Karlsen S Nazroo JY Relation between racial discrimination, social class, and health among ethnic minority groups Am J Public Health 2002 92 624 631 10.2105/AJPH.92.4.624 11919063 PMC1447128

[B7] Coker TR Elliott MN Kanouse DE Grunbaum JA Schwebel DC Gilliland MJ Tortolero SR Peskin MF Schuster MA Perceived racial/ethnic discrimination among fifth-grade students and its association with mental health Am J Public Health 2009 99 878 884 10.2105/AJPH.2008.144329 19299673 PMC2667854

[B8] Virdee S Madood T, Berthoud R, Lakey J, Nazroo J, Smith P, Virdee S, Beishon S Racial harassment Ethnic minorities in Britain: Diversity and disadvantage 1997 London: Policy Studies Institute 259 289

[B9] Nazroo JY The structuring of ethnic inequalities in health: economic position, racial discrimination, and racism Am J Public Health 2003 93 277 284 10.2105/AJPH.93.2.277 12554585 PMC1447729

[B10] Larson A Gillies M Howard P Coffin J It's enough to make you sick: the impact of racism on the health of Aboriginal Australians Aust N Z J Public Health 2007 31 322 10.1111/j.1753-6405.2007.00079.x 17725009

[B11] Mays VM Cochran SD Barnes NW Race, race-based discrimination, and health outcomes among African Americans Annu Rev Psychol 2007 58 201 225 10.1146/annurev.psych.57.102904.190212 16953796 PMC4181672

[B12] Stuber J Galea S Ahern J Blaney S Fuller C The Association between Multiple Domains of Discrimination and Self-assessed Health: A Multilevel Analysis of Latinos and Blacks in Four Low-Income New York City Neighborhoods Health Serv Res 2003 38 1735 1760 10.1111/j.1475-6773.2003.00200.x 14727795 PMC1360971

[B13] Priest N Paradies Y Stewart P Luke J Racism and health among urban Aboriginal young people BMC Public Health 2011 11 568 10.1186/1471-2458-11-568 21756369 PMC3146875

[B14] Williams DR Mohammed SA Discrimination and racial disparities in health: evidence and needed research J Behav Med 2009 32 20 47 10.1007/s10865-008-9185-0 19030981 PMC2821669

[B15] Finch BK Kolody B Vega WA Perceived discrimination and depression among Mexican-origin adults in California J Health Soc Behav 2000 41 295 313 10.2307/2676322 11011506

[B16] Bowen-Reid TL Harrell JP Racist experiences and health outcomes: an examination of spirituality as a buffer J Black Psychol 2002 28 18 36 10.1177/0095798402028001002

[B17] Shavers VL Shavers BS Racism and health inequity among Americans J Natl Med Assoc 2006 98 386 396 16573303 PMC2576116

[B18] Dotterer AM McHale SM Crouter AC Sociocultural factors and school engagement among African American youth: the roles of racial discrimination, racial socialization, and ethnic identity Appl Dev Sci 2009 13 61 73 10.1080/10888690902801442 PMC4850833 27134516

[B19] Clark R Anderson NB Racism as a stressor for African Americans Am Psychol 1999 54 805 10540593 10.1037//0003-066x.54.10.805

[B20] Paradies Y A systematic review of empirical research on self-reported racism and health Int J Epidemiol 2006 35 888 901 10.1093/ije/dyl056 16585055

[B21] Pachter LM Coll CG Racism and child health: a review of the literature and future directions J Dev Behav Pediatr 2009 30 255 263 10.1097/DBP.0b013e3181a7ed5a 19525720 PMC2794434

[B22] Yoo HC Gee GC Lowthrop CK Robertson J Self-reported racial discrimination and substance use among Asian Americans in Arizona J Immigr Health Minor Health 2010 12 683 690 10.1007/s10903-009-9306-z 20012204

[B23] Surko M Ciro D Blackwood C Nembhard M Peake K Experience of racism as a correlate of developmental and health outcomes among urban adolescent mental health clients Soc Work Ment 2005 3 235 260 10.1300/J200v03n03_02

[B24] Harris R Tobias M Jeffreys M Waldegrave K Karlsen S Nazroo J Effects of self-reported racial discrimination and deprivation on Maori health and inequalities in New Zealand: cross-sectional study Lancet 2006 367 2005 2009 10.1016/S0140-6736(06)68890-9 16782491

[B25] Denny SJ Grant S Utter J Robinson EM Fleming TM Milfont TL Crengle S Clark T Ameratunga SN Dixon R Health and well-being of young people who attend secondary school in Aotearoa, New Zealand: What has changed from 2001 to 2007? J Paediatr Child Health 2011 47 191 197 10.1111/j.1440-1754.2010.01945.x 21244550

[B26] Robson B Purdie G Robson B, Harris R Mortality Hauora: Māori Standards of Health IV A study of the years 2000-2005 2007 Wellington: Te Rōpū Rangahau a Eru Pōmare and University of Otago17339900

[B27] Robson B Robson C Harris R Purdie G Robson B, Harris R Hospitalisations Hauora: Māori Standards of Health IV A study of the years 2000-2005 2007 Wellington: Te Rōpū Rangahau a Eru Pōmare and University of Otago17339900

[B28] Clark TC Robinson E Crengle S Herd R Grant S Denny S Te Ara Whakapiki Taitamariki Youth'07- the Health and Wellbeing of Secondary School Students in New Zealand. Results for Māori young People Book Te Ara Whakapiki Taitamariki Youth'07- the Health and Wellbeing of Secondary School Students in New Zealand. Results for Māori young People 2008 Auckland: University of Auckland

[B29] Helu S Robinson E Grant S Herd R Denny S Youth '07 - the Health and Wellbeing of Secondary School Students in New Zealand Results for Pacific Young People Book Youth '07 - the Health and Wellbeing of Secondary School Students in New Zealand Results for Pacific Young People 2009 Auckland: University of Auckland22259070

[B30] Adolescent Health Research Group Youth'07: The Health and Wellbeing of Secondary School Students in New Zealand. Technical Report Book Youth'07: The Health and Wellbeing of Secondary School Students in New Zealand. Technical Report 2008 Auckland: The University of Auckland22259070

[B31] Denny S Milfont T Utter J Robinson E Ameratunga S Merry S Fleming T Watson P Hand-held internet tablets for school-based data collection BMC Research Notes 2008 1 52 10.1186/1756-0500-1-52 18710505 PMC2525643

[B32] Statistical Standard for Ethnicity 2005 http://www2.stats.govt.nz/domino/external/web/carsweb.nsf/55d63ae38ba3a25e4c2567e6007f6686/35d9b7e17a1d6151cc25701100031353?OpenDocument

[B33] Ministry of Health Ethnicity Data Protocols for the Health and Disability Sector Book Ethnicity Data Protocols for the Health and Disability Sector 2004 Wellington: Ministry of Health

[B34] Salmond C Crampton P Atkinson J NZDep2006 Index of Deprivation Book NZDep2006 Index of Deprivation 2007 Wellington: Department of Public Health, University of Otago

[B35] Reynolds WM Mazza JJ Reliability and validity of the reynolds adolescent depression scale with young adolescents J Sch Psychol 1998 36 295 10.1016/S0022-4405(98)00010-7

[B36] Walker L Merry S Watson PD Robinson E Crengle S Schaaf D The Reynolds adolescent depression scale in New Zealand adolescents Aust N Z J Psychiatry 2005 39 136 140 10.1080/j.1440-1614.2005.01534.x 15701061

[B37] Milfont TL Merry S Robinson E Denny S Crengle S Ameratunga S Evaluating the short form of the Reynolds adolescent depression scale in New Zealand adolescents Aust N Z J Psychiatry 2008 42 950 954 10.1080/00048670802415343 18941959

[B38] Smedley BD Stith AY Nelson AR Unequal treatment: Confronting racial and ethnic disparities in health care 2003 Washington DC: The National Academies Press 25032386

[B39] van Ryn M Burke J The effect of patient race and socio-economic status on physicians' perceptions of patients Soc Sci Med 2000 50 813 828 10.1016/S0277-9536(99)00338-X 10695979

[B40] LaVeist TA Rolley NC Diala C Prevalence and patterns of discrimination among U.S. health care consumers Int J Health Serv 2003 33 331 344 10.2190/TCAC-P90F-ATM5-B5U0 12800890

[B41] Blanchard J Lurie N R-E-S-P-E-C-T: patient reports of disrespect in the health care setting and its impact on care J Fam Pract 2004 53 721 730 15353162

[B42] Seaton EK Caidwell CH Sellers RM Jackson JS The prevalence of perceived discrimination among African American and Caribbean black youth Dev Psychol 2008 44 1288 1297 18793063 10.1037/a0012747 PMC2556985

[B43] Wortley S Tanner J Discrimination or 'good' policing? The racial profiling debate in Canada Our Diverse Cities 2004 1 197 201

[B44] Rosenbloom SR Way N Experiences of discrimination among African American, Asian American, and Latino Adolescents in an urban high school Youth & Soc 2004 35 420 45122262916

[B45] Fleming T Watson P Robinson E Ameratunga S Dixon R Clark T Crengle S Violence and New Zealand young people: findings of youth2000 - a national secondary school youth health and wellbeing survey Book Violence and New Zealand young people: Findings of Youth2000 - A national secondary school youth health and wellbeing survey 2007 Auckland: University of Auckland

[B46] Ministry of Education The experiences of international students in New Zealand Book The experiences of international students in New Zealand 2004 Wellington: The Ministry of Education

[B47] Sobrun-Maharaj A The social acceptance of visible ethnic minority adolescents of Asian origin in Auckland secondary schools Doctor of Philosophy 2002 Massey University

[B48] Ameratunga S Horner J 'Asian' and immigrant minority youth in Aotearoa/New Zealand Improving the Transition Reducing the social and psychological morbidity during adolescence A report from the Prime Minister's Chief Scientific Advisor 2011 Wellington, New Zealand: Office of the Prime Minister's Science Advisory Committee

[B49] Fisher CB Wallace SA Discrimination distress during adolescence J Youth Adolesc 2000 29 679 10.1023/A:1026455906512

[B50] Eslea M Mukhtar K Bullying and racism among Asian schoolchildren in Britain Educational Research 2000 42 207 217 10.1080/001318800363845

[B51] Williams DR Neighbors HW Jackson JS Racial/ethnic discrimination and health: findings from community studies Am J Public Health 2003 93 200 208 10.2105/AJPH.93.2.200 12554570 PMC1447717

[B52] Santana V Almeida-Filho N Roberts R Cooper SP Skin colour, perception of racism and depression among adolescents in Urban Brazil Child Adolesc Ment Health 2007 12 125 131 10.1111/j.1475-3588.2007.00447.x 32811081

[B53] Brody GH Yi-Fu C Murry VM Simons RL Xiaojia G Gibbons FX Gerrard M Cutrona CE Perceived discrimination and the adjustment of African American youths: a five-year longitudinal analysis with contextual moderation effects Child Dev 2006 77 1170 1189 10.1111/j.1467-8624.2006.00927.x 16999791

[B54] Szalacha LA Erkut S García Coll C Alarcón O Fields JP Ceder I Discrimination and Puerto Rican children's and adolescents' mental health Cultur Divers Ethni Minor Psychol 2003 9 141 155 10.1037/1099-9809.9.2.141 12760326

[B55] Pascoe EA Smart Richman L Perceived discrimination and health: a meta-analytic review Psychol Bull 2009 135 531 554 19586161 10.1037/a0016059 PMC2747726

[B56] Bennett GG Wolin KY Robinson EL Fowler S Edwards CL Perceived racial/ethnic harassment and tobacco use among African American young adults Am J Public Health 2005 95 238 240 10.2105/AJPH.2004.037812 15671457 PMC1449159

[B57] Landrine H Klonoff EA Racial discrimination and cigarette smoking among blacks: findings from two studies Ethn Dis 2000 10 195 202 10892825

[B58] Chae DH Takeuchi DT Barbeau EM Bennett GG Lindsey J Krieger N Unfair treatment, racial/ethnic discrimination, ethnic identification, and smoking among Asian Americans in the National Latino and Asian American Study Am J Public Health 2008 98 485 492 10.2105/AJPH.2006.102012 18235073 PMC2253562

[B59] Nansel TR Overpeck MD Haynie DL Ruan WJ Scheidt PC Relationships between bullying and violence among US youth Arch Pediatr Adolesc Med 2003 157 348 353 10.1001/archpedi.157.4.348 12695230

[B60] Glover D Gough G Johnson M Cartwright N Bullying in 25 secondary schools: incidence, impact and intervention Educ Res 2000 42 141 156 10.1080/001318800363782

[B61] Spriggs AL Iannotti RJ Nansel TR Haynie DL Adolescent bullying involvement and perceived family, peer and school relations: commonalities and differences across race/ethnicity J Adolesc Health 2007 41 283 293 10.1016/j.jadohealth.2007.04.009 17707299 PMC1989108

[B62] Bals M Turi AL Skre I Kvernmo S The relationship between internalizing and externalizing symptoms and cultural resilience factors in Indigenous Sami youth from Arctic Norway Int J Circumpolar Health 2011 70 37 45 21329576 10.3402/ijch.v70i1.17790

[B63] Guttmacher S Weitzman BC Kapadia F Weinberg SL Classroom-based surveys of adolescent risk-taking behaviors: reducing the bias of absenteeism Am J Public Health 2002 92 235 237 10.2105/AJPH.92.2.235 11818298 PMC1447049

[B64] Stone S Han M Perceived school environments, perceived discrimination, and school performance among children of Mexican immigrants Child Youth Serv Rev 2005 27 51 66 10.1016/j.childyouth.2004.08.011

[B65] Stand downs, suspensions, exclusions and expulsions from school http://www.educationcounts.govt.nz/indicators/main/student-engagement-participation/80346

[B66] Robson B Cormack D Cram F Robson B, Harris R Social and economic indicators Hauora: Māori standards of health IV A study of the years 2000-2005 2007 Wellington: Te Rōpū Rangahau Hauora a Eru Pōmare17339900

[B67] Wardle J Robb K Johnson F Assessing socioeconomic status in adolescents: the validity of a home affluence scale J Epidemiol Community Health 2002 56 595 599 10.1136/jech.56.8.595 12118050 PMC1732226

[B68] Currie CE Elton RA Todd J Platt S Indicators of socioeconomic status for adolescents: the WHO health behaviour in school-aged children survey Health Educ Res 1997 12 385 397 10.1093/her/12.3.385 10174221

